# Anterior Cricoid Abscess with a Posterior Granuloma: A Dual Pathology

**DOI:** 10.22038/ijorl.2025.80509.3707

**Published:** 2025

**Authors:** Pei Fen Cheah, Sien Hui Tan

**Affiliations:** 1 *Department of Otorhinolaryngology, Faculty of Medicine, Universiti Malaya, Kuala Lumpur, Malaysia.*

**Keywords:** Abscess, Cricoid cartilage, Hoarseness, Stridor, Granuloma

## Abstract

**Introduction:**

Cricoid abscesses are rare, typically occurring in the posterior part of the cricoid and are often life-threatening. In this paper, we describe the first reported case of an anterior cricoid abscess and discuss our management.

**Case Report:**

A 60-year-old lady presented with dyspnoea for 2 days and progressive hoarseness for 8 months. A contrast-enhanced computed tomography revealed a posterior soft tissue thickening and an anterior subglottic collection. The patient underwent endoscopic excision of the granuloma and drainage of the abscess. She completed 3 weeks of oral ciprofloxacin. Subsequent follow-up revealed a patent airway.

**Conclusion:**

We were highly suspicious, as stridor is unlikely to be caused solely by a posterior granuloma. Clinicians should remain alert to the possibility of a second pathology, and early imaging should be performed when clinically indicated. The rarity of a cricoid abscess necessitates a high index of suspicion for diagnosis. In this case, progressive laryngeal lumen narrowing caused by posterior granuloma and cricoid abscess facilitated early symptom detection and timely management, thereby averting tracheostomy.

## Introduction

Cricoid abscesses are exceedingly rare. These infections typically occur in the posterior part of the cricoid and are often life-threatening. Urgent investigation and management are crucial to prevent airway compromise and limit potential complications. In this paper, we describe the first case of an anterior cricoid abscess and discuss our experience in the management of this patient. 

## Case Reports

A 60-year-old lady presented to the emergency department with a 2-day history of dyspnoea, and progressive hoarseness for 8 months. She was previously intubated for 11 days following an emergency sternotomy due to an iatrogenic right ventricle perforation during a permanent pacemaker insertion 10 months ago. Her medical history includes end-stage renal failure and hypertension. Clinical examination revealed biphasic stridor, but she was otherwise afebrile and oxygen saturation was 100% under room air. Flexible nasopharyngolaryngoscopy demonstrated a posterior granuloma with subglottic edema. Her vocal cords were mobile and her supraglottic structures were unremarkable. 

**Fig 1:**
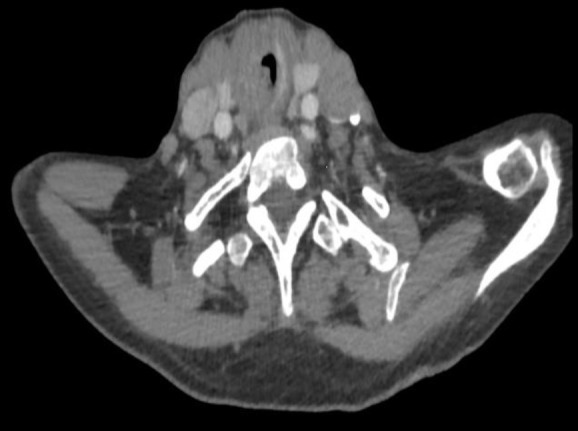
Axial view of contrast-enhanced computed tomography showing a posterior non-enhancing isodense soft tissue thickening.

Intravenous dexamethasone, antibiotics and pantoprazole were initiated. Laboratory investigations, including inflammatory markers, were unremarkable, but her condition did not improve. Contrast-enhanced computed tomography demonstrated a non-enhancing isodense soft tissue thickening at the posterior subglottic region, resulting in significant airway stenosis (Figure 1). 

**Fig 2:**
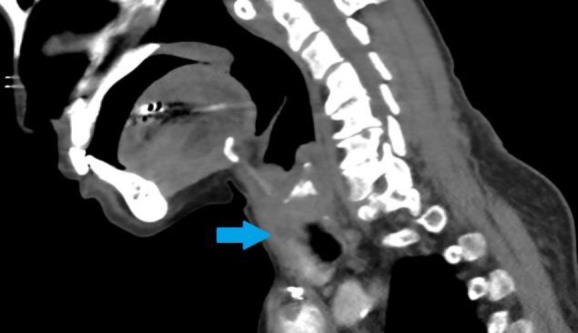
Sagittal view of contrast-enhanced computed tomography showing a small anterior subglottic collection (blue arrow).

Interestingly, there was an unexpected finding of a small anterior subglottic collection with associated inflammatory changes surrounding the right antero-lateral cricoid cartilage (Figure 2). 

**Fig 3:**
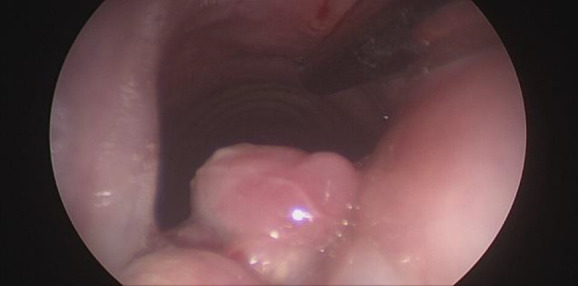
Endoscopic view of posterior granuloma and edematous anterior cricoid mucosa.

The patient was brought to the operating room and successfully intubated with a 6-0 mm cuffed endotracheal tube. Suspension laryngoscopy revealed a posterior granuloma extending inferiorly from the vocal cord and an edematous anterior cricoid mucosa which was fluctuant on palpation (Figure 3). 

**Fig 4:**
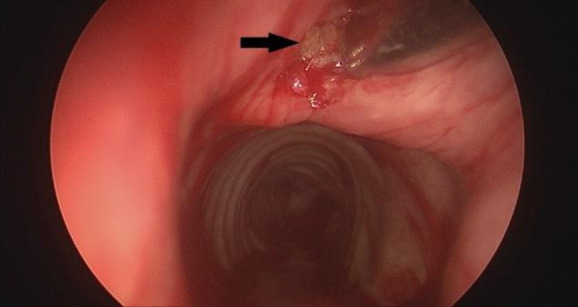
Post endoscopic drainage showing sequestrum of cricoid cartilage (black arrow).

Endoscopic excision of the posterior granuloma and drainage of 2 mL of purulent fluid from the anterior cricoid abscess were performed, revealing an underlying sequestrum of cricoid cartilage (Figure 4).

The patient was extubated uneventfully and showed marked clinical improvement. Flexible nasopharyngolaryngoscopy demonstrated resolving subglottic oedema. Culture isolated Pseudomonas aeruginosa, and the antibiotic regimen was adjusted accordingly. The histopathology of the anterior subglottic wall was consistent with an abscess wall. The patient was discharged well 5 days post-operatively with a 3-week course of oral ciprofloxacin. Subsequent follow-up revealed a patent airway with no subglottic oedema. 

## Discussion

Although cricoid abscesses are exceedingly uncommon, they carry a high risk of morbidity and mortality owing to potential airway obstruction. All previously documented cases have described abscesses arising from the posterior aspect of the cricoid cartilage (1-5). To the best of our knowledge, this is the first reported case involving the anterior cricoid region.

The pathophysiology of cricoid abscesses remains poorly understood. Several authors have postulated that abscess formation may result from laryngeal perichondritis secondary to mucosal injury after prolonged intubation (2,4). In this case, the initial intubation trauma was likely extensive, following an emergency sternotomy, leading to the ensuing posterior granuloma. We further postulate that pressure necrosis caused by the endotracheal tube cuff led to perichondritis, as evidenced by the inflammatory changes in the right anterolateral cricoid cartilage, predisposing the area to abscess formation. 

Additionally, an impaired ability to produce an appropriate immunologic response appears to facilitate the process for laryngeal abscesses (3). Diabetes mellitus leads to dysfunction of the immune response and has been reported in patients with cricoid abscesses (1,2). Similarly, for our patient with end stage renal failure, her immunocompromised state likely increased her susceptibility to a cricoid abscess formation. Conversely, gastroesophageal reflux may play a role in the pathogenesis of both cricoid abscess and posterior granuloma. 

We believe that the cricoid abscess and the posterior granuloma developed independently of each other. Posterior granuloma was formed as a result of an exaggerated healing process in response to the extensive intubation injury. Therefore, these pathologies are two separate entities and have no direct causal relationship in this case. The interval between trauma and presentation of symptoms is variable in immunocompromised patients. Souliere et al (1) reported the presentation as early as 2 to 4 days after nasogastric tube placement for their patient with diabetes. Interestingly, our patient had a very delayed presentation at 10 months post intubation.

The clinical course for cricoid abscesses is rather insidious. Patients may be afebrile and present with progressive hoarseness (2,4). They become symptomatic only when the laryngeal lumen narrows, thus, there is a risk for late diagnosis. Delayed diagnosis significantly increases the likelihood of advanced disease. In cricoid abscesses, the resultant collection is typically extensive and may cause imminent airway obstruction. 

Although our patient also presented in a similar manner, her lumen was already narrow due to the posterior granuloma. Hence, additional obstruction from the subglottic oedema and anterior cricoid abscess led to earlier onset of symptoms, allowing detection at a stage when the collection remained small. It is important to emphasize that the prominent appearance of the posterior granuloma could have easily obscured the underlying diagnosis. In our case, we remained highly suspicious, since stridor alone could not be explained by the posterior granuloma, prompting further evaluation with imaging. 

Hence, clinicians should always be mindful of the possibility of a second pathology. This case represents a rare and noteworthy example of dual pathology involving the larynx. 

Treatment modalities include endoscopic incision and drainage with antibiotics as well as tracheostomy for advanced airway compromise. In nearly all the cases reported, a tracheostomy was necessary to relieve airway obstruction (1,2,4,5).

 For our patient, we did not perform a tracheostomy as the collection was small and we were confident in the complete drainage of the abscess collection. The principal advantage of this approach lies in minimizing further damage to the existing laryngeal framework and avoiding morbidity associated with tracheostomy. 

Nevertheless, one should be aware of the possibility of oedema developing post-operatively and the risk of recollection of abscess leading to airway compromise. Additionally, surveillance of subglottic area during clinic follow-up may be difficult and can induce laryngospasm in some patients. In such cases, examination under general anaesthesia may be required for adequate reassessment.

## Conclusion

The rarity of a cricoid abscess necessitates a high index of suspicion for diagnosis. In our case, the coexistence of a posterior granuloma and an anterior cricoid abscess highlights the importance of considering dual pathology in patients with atypical presentations. 

Clinicians should maintain vigilance for a second underlying pathology when symptoms cannot be fully explained by a single finding. Prompt recognition and management are essential to prevent airway compromise, and early diagnosis may allow avoidance of tracheostomy and its associated morbidity.

### Declaration of Conflicting Interests:

The author(s) declare(s) that there is no conflict of interest. 

### Funding

The author(s) received no financial support for the research, authorship, and/or publication of this article.
